# Supplementary material for: Analysis of PPARγ Signaling Activity in Psoriasis
Source: Int J Mol Sci. 2021 Aug 10;22(16):8603. doi: 10.3390/ijms22168603 (PMC8395241; doi:10.3390/ijms22168603)
Supplement: Supplementary file 1 [file ijms-22-08603-s001.zip › Supplemental materials_Analysis of PPARg signaling activity in psoriasis/Pathway models/Models images and html files/PPARG expressed in cells assotiated with psoriasis/225990.html]

mast cell


# CellType mast cell

|  |  |
| --- | --- |
| URN | urn:agi-ncimcelltype:C0024880 |
| Total Entities | 61 |
| Connectivity | 5582 |
| Name | mast cell |

---

|  |  |
| --- | --- |
| ChildConcepts | mast cell adhesion |
|  | mast cell chemotaxis |
|  | mast cell homeostasis |
|  | mast cell apoptosis |
|  | mast cell differentiation |
|  | mast cell proliferation |
|  | bone marrow derived mast cell |
|  | peritoneum mast cell |
|  | mast cell infiltration |
|  | mast cell phenotype |
|  | mast cell migration |
|  | mast cell function |
|  | mast cell development |
|  | mast cell distribution |
|  | mast cell survival |
|  | mast cell localization |
|  | mast cell growth |
|  | mast cell division |
|  | mast cell death |
|  | mast cell motility |
|  | mast cell mediated cytotoxicity |
|  | mast cell homing |
|  | mast cell aggregation |
|  | mast cell priming |
|  | mast cell population |
|  | mast cell formation |
|  | mast cell viability |
|  | mast cell interaction |
|  | mast cell phagocytosis |
|  | mast cell proliferative response |
|  | mast cell swelling |
|  | mast cell repertoire |
|  | mast cell structure |
|  | mast cell size |
|  | mast cell renewal |
|  | mast cell spreading |
|  | mast cell motion |
|  | mast cell stress |
|  | mast cell macrophage differentiation |
|  | mast cell sequestration |
|  | mast cell fate commitment |
|  | mast cell extravasation |
|  | mast cell fate |
|  | mast cell volume |
|  | mast cell clonal anergy |
|  | mast cell disruption |
|  | mast cell damage |
|  | mast cell communication |
|  | mast cell recognition |
|  | mast cell invasion |
|  | mast cell cytotoxicity |
|  | mast cell polarity |
|  | mast cell lifespan |
|  | mast cell destruction |
|  | mast cell contact |
|  | mast cell fate determination |
|  | mast cell transendothelial migration |
|  | mast cell anchorage |
|  | mast cell selection |
|  | mast cell ultrastructure |
|  | mast cell regeneration |

---

|  |  |
| --- | --- |
| Pathway | PPARG expressed in cells assotiated with psoriasis |
|  | Neighbors of mast cell |
|  | 2\_3\_Eosinophilia and neutrophilia in asthma: Neutrophil chemotaxis and activation |

---

|  |  |
| --- | --- |
| MedScan ID | 10003407 |

---

|  |  |
| --- | --- |
| Alias | labrocytes |
|  | histaminocytes |
|  | mast cells |
|  | tissue basophil |
|  | tissue basophils |
|  | labrocytic |
|  | mastocytes |
|  | histaminocyte |
|  | labrocyte |
|  | mastocyte |
|  | mast cell |

---

|  |  |
| --- | --- |
| NCIm ID | C0024880 |

---
